# Pic2Plate: A Vision-Language and Retrieval-Augmented Framework for Personalized Recipe Recommendations

**DOI:** 10.3390/s25020449

**Published:** 2025-01-14

**Authors:** Yosua Setyawan Soekamto, Andreas Lim, Leonard Christopher Limanjaya, Yoshua Kaleb Purwanto, Suk-Ho Lee, Dae-Ki Kang

**Affiliations:** 1 Department of Computer Engineering, Dongseo University, Busan 47011, Republic of Korea; yosua.soekamto@ciputra.ac.id (Y.S.S.); andreaslim2002@gmail.com (A.L.); leonardchristopher002@gmail.com (L.C.L.); yoshuakaleb049@gmail.com (Y.K.P.); petra@g.dongseo.ac.kr (S.-H.L.); 2 Department of Information Systems, Universitas Ciputra Surabaya, Surabaya 60219, Indonesia

**Keywords:** retrieval-augmented generation, personalized recipe recommendation, large language models, vision-language models, ingredient-based recipe retrieval

## Abstract

Choosing nutritious foods is essential for daily health, but finding recipes that match available ingredients and dietary preferences can be challenging. Traditional recommendation methods often lack personalization and accurate ingredient recognition. Personalized systems address this by integrating user preferences, dietary needs, and ingredient availability. This study presents Pic2Plate, a framework combining Vision-Language Models (VLMs) and Retrieval-Augmented Generation (RAG) to overcome these challenges. Pic2Plate uses advanced image recognition to extract ingredient lists from user images and RAG to retrieve and personalize recipe recommendations. Leveraging smartphone camera sensors ensures accessibility and portability. Pic2Plate’s performance was evaluated in two areas: ingredient detection accuracy and recipe relevance. The ingredient detection module, powered by GPT-4o, achieved strong results with precision (0.83), recall (0.91), accuracy (0.77), and F1-score (0.86), demonstrating effectiveness in recognizing diverse food items. A survey of 120 participants assessed recipe relevance, with model rankings calculated using the Bradley–Terry method. Pic2Plate’s VLM and RAG integration consistently outperformed other models. These results highlight Pic2Plate’s ability to deliver context-aware, reliable, and diverse recipe suggestions. The study underscores its potential to transform recipe recommendation systems with a scalable, user-centric approach to personalized cooking.

## 1. Introduction

The importance of balanced and nutritious meals has been consistently linked to physical and mental health benefits. Diets rich in essential nutrients, such as omega-3 fatty acids, B vitamins, and minerals like zinc, have shown a positive impact on mental health outcomes, helping reduce symptoms of depression, anxiety, and cognitive decline. The research underscores that the regular intake of nutrient-dense foods can support neurotransmitter function, neuroplasticity, and anti-inflammatory processes, contributing to overall well-being [1]. However, creating such meals regularly is challenging for many, particularly due to time constraints and decision fatigue, both of which hinder people’s ability to make healthy, fulfilling meal choices efficiently.

Once people settle on a meal choice, aligning available ingredients with chosen recipes introduces additional hurdles. The limited availability of specific ingredients frequently results in the need for substitutions or even the abandonment of the recipe altogether [2,3,4]. Studies show that many individuals find it difficult to identify or use ingredients effectively, particularly when the ingredient names are unfamiliar or less commonly used. A recommendation system can help by suggesting recipes that align more closely with the resources users already have, minimizing the need for substitutions [5,6]. This issue is further complicated by the fact that conventional recipe recommendations often do not take into account individual constraints or unique preferences, leaving users with recipes that may not align with their available ingredients or dietary needs. These obstacles highlight a critical gap in current recommendation systems, emphasizing the need for solutions that dynamically adapt to user-specific ingredient inventories and dietary requirements [2,3,7].

Personalization in recipe recommendation systems remains a complex task, as users have distinct dietary needs, preferences, and culinary skill levels. Factors like food allergies, dietary restrictions, and flavor preferences introduce layers of complexity that current systems often fail to address comprehensively. This lack of nuanced personalization can lead to confusion in the decision-making process, where users are overwhelmed by numerous recipe options but struggle to find one that matches their specific needs [8]. For individuals with health-based dietary requirements, a system that can provide recommendations aligned with their unique dietary profile could significantly enhance meal satisfaction and support adherence to health goals.

In response to these issues, the development of advanced recipe recommendation systems has become a key area of interest. Recent approaches have integrated machine learning and image recognition to better align recipe suggestions with available ingredients and user preferences. While text-based systems rely heavily on explicit user input, image-based systems attempt to bridge this gap by recognizing ingredients visually, thereby enabling recommendations based on physical availability. However, image detection-based frameworks often depend on large, domain-specific datasets to build their knowledge base, requiring extensive data collection and curation to achieve acceptable performance levels. This dependency on massive datasets posed challenges in terms of scalability, diversity, and adaptability to new scenarios [9,10]. Additionally, text-based methods can be restrictive, as users may lack clarity on desired recipes, and image-based systems often fail to accommodate dietary nuances fully. These limitations highlight the need for hybrid systems that merge the strengths of multiple data sources is becoming increasingly apparent, as such systems could adapt dynamically to the evolving dietary needs and preferences of users.

The growing need for personalized recipe recommendation systems comes from the desire to make meal planning easier and more accessible [11]. While existing systems have made strides in offering tailored suggestions, they often struggle to address individual constraints such as limited ingredient availability, specific dietary needs, or personal flavor preferences. For instance, a user with only a few vegetables in their refrigerator may find it difficult to identify recipes that match both their ingredients and dietary restrictions. Smartphones, equipped with high-resolution cameras, have emerged as essential tools for these systems, enabling users to seamlessly capture ingredient images for real-time processing. This integration of convenience and technology has created opportunities for frameworks that can analyze both visual and textual data to provide practical, user-focused solutions.

Vision-Language Models (VLMs) and Retrieval-Augmented Generation (RAG) represent pivotal advancements in this domain. VLMs enable systems to understand and process multimodal data, such as ingredient images and dietary prompts, offering a nuanced approach to contextual analysis [12,13,14,15]. For instance, a VLM can identify specific ingredients in an image while simultaneously interpreting user instructions for recipe constraints, such as “low-carb” or “gluten-free”. This ability to interpret both visual and language data makes VLMs an ideal foundation for applications that require nuanced understanding, such as matching recipes to available ingredients or aligning suggestions with dietary needs. However, despite their capabilities, VLMs alone may struggle to generate precise recommendations, particularly in scenarios where additional contextual information or specific user constraints, such as allergies or substitutions, must be considered [16].

RAG frameworks address these challenges by integrating a retrieval mechanism that supplements generative outputs with information from structured databases [17,18]. In the context of recipe recommendations, when a user provides an image of mixed vegetables and specifies a vegetarian preference, RAG enhances the system’s ability to retrieve recipes that not only match these ingredients but also adhere to the dietary prompt. This layered approach ensures that the recommended recipes not only match user expectations but also maintain coherence with the available ingredients, dietary restrictions, and culinary skills, ultimately bridging the gap between user input and model output.

To address these challenges, we propose a recipe recommendation framework called Pic2Plate, which combines the strengths of VLMs and RAG to deliver highly personalized, context-aware recipe suggestions. Unlike conventional systems, Pic2Plate dynamically adapts to user inputs by analyzing ingredient images alongside user-defined dietary preferences or prompts. Additionally, it incorporates an always-up-to-date recipe dataset, retrieved from online sources, to ensure that the recommendations remain relevant and reflect the latest culinary trends. By bridging the gap between dietary goals and practical meal solutions, Pic2Plate offers a seamless, user-friendly experience that simplifies meal planning while meeting individual needs.

Our contributions to this work are as follows:

- A Novel Recipe Recommendation System: We introduce Pic2Plate, a recipe recommendation framework that combines VLM and RAG. Pic2Plate processes both ingredient images and user preferences to recommend relevant recipes.
- Enhanced RAG with Online Retrieval: We improve the RAG framework by incorporating an online retrieval mechanism, allowing the system to continuously update its recipe database with newly available data, ensuring that Pic2Plate remains relevant and accurate over time.
- Evaluation of Pic2Plate’s Performance: We demonstrate the effectiveness of Pic2Plate by analyzing ingredient detection accuracy in image segmentation, as well as evaluating the recommendation accuracy in matching recipes to user preferences and ingredient inputs. The image segmentation module was tested using a mix of images captured with smartphone cameras and images sourced from the internet, ensuring robustness across diverse input types.

## 2. Related Work

### 2.1. Vision-Language Models

Image segmentation is a foundational technique in computer vision that involves partitioning an image into distinct regions or segments, each representing meaningful parts of the image. This process is crucial for isolating specific objects within an image, enabling further analysis of each segment [19,20]. Segmentation methods range from traditional approaches, such as thresholding, region-based segmentation, and edge detection, to more advanced deep learning techniques. While traditional methods use pixel intensity differences to delineate regions, deep learning-based methods leverage Convolutional Neural Networks (CNNs) to learn complex features, making them more robust for handling diverse, real-world images.

Deep learning methods, such as U-Net, Mask R-CNN, and DeepLab, have become the standard in image segmentation due to their ability to learn from large datasets and adapt to complex visual patterns. These models are especially effective in identifying overlapping objects and recognizing textures and shapes, which are common in food images. However, deep learning segmentation models are resource intensive and may struggle with certain real-world challenges, like occlusion and varied lighting, which can affect their accuracy. Recent advancements have sought to address these limitations, introducing improvements like attention mechanisms and multi-scale learning to enhance segmentation performance [21,22,23,24,25,26].

One key application of image segmentation is ingredient recognition, where segmentation techniques are used to identify and isolate specific food items within an image. Applying image segmentation to ingredient recognition introduces additional complexities, as food items often vary in appearance and may overlap within the image [24,27,28,29]. Ingredient recognition requires the precise segmentation of individual items, often involving tailored approaches that account for ingredient-specific challenges, such as texture and color similarity. Baseline methods for ingredient recognition frequently rely on CNNs trained on food image datasets (e.g., Food-101 or Recipe1M), achieving high accuracy in controlled settings. However, these models may underperform with real-world food images, where ingredients can appear mixed or partially obscured.

Recent studies have aimed to enhance ingredient recognition by adopting multi-scale architectures and label dependency learning, as seen in models like FoodNet, which incorporates label dependencies to improve segmentation accuracy for food items. Other advancements include the use of task-specific modifications, such as single-ingredient classification models that improve segmentation in complex food scenes. These models heavily depend on large and diverse datasets to build robust and reliable models, as the variability in ingredient appearance, preparation states, and environmental factors requires comprehensive training data [9,10]. Such models demonstrate progress in making ingredient recognition more adaptable to diverse food images, bridging a gap between traditional image segmentation and practical applications in food analysis.

Vision-Language Models (VLMs) represent a newer class of models that integrate visual and textual data, allowing them to process images while understanding the corresponding descriptive language. A newer approach to image segmentation leverages this integration with language models, creating Vision-Language Models capable of understanding both visual details and context from language inputs [13,30,31]. VLMs like Contrastive Language-Image Pretraining (CLIP) and Bootstrapped Language-Image Pretraining (BLIP), are pretrained on paired image–text data, enabling them to generate captions, answer questions, and even recommend items based on visual input [32,33]. These models use architectures that combine CNNs or transformers for image processing with language models, facilitating a dual understanding of images and text. This integration makes VLMs especially useful in applications requiring contextual awareness, such as matching ingredient images to recipe descriptions [12].

In the context of ingredient recognition and recipe recommendations, VLMs offer a promising solution by interpreting visual cues and matching them with relevant textual information. However, current VLMs may struggle with tasks requiring fine-grained ingredient recognition, as they often generalize visual information rather than segmenting and labeling each component precisely. Recent research has aimed to enhance VLMs by incorporating Retrieval-Augmented Generation (RAG) frameworks and leveraging large datasets to improve model accuracy and contextual understanding, making them more effective for practical applications in food recognition and recipe recommendation [16].

### 2.2. Recipe Recommendation System (Textual/Tabular Dataset to Recipe Recommendation)

Recommendation systems are designed to suggest relevant items to users based on their preferences or past interactions, and they play an essential role in personalizing user experiences across many applications [11]. Traditional recommendation systems primarily rely on structured data, often stored in tables or databases, where items are recommended based on collaborative filtering, content-based filtering, or hybrid approaches. In the context of food, recommendation systems typically use textual or tabular recipe datasets that include information on ingredients, preparation steps, and descriptions, enabling them to match user inputs with relevant recipe suggestions.

Traditional methods also include rule-based systems, where recipes are suggested based on predefined conditions, such as selecting a dish type or excluding allergens. Some systems focus on health and nutrition, recommending recipes based on dietary goals like low-carb, high-protein, or low-sodium diets. Additionally, user-generated content—such as reviews, ratings, and feedback—has been used to refine suggestions by incorporating real-time user insights [34,35,36]. While these systems have improved personalization to some extent, they still struggle with real-time ingredient availability, complex dietary needs, and dynamic user preferences, highlighting the need for more adaptive and integrative solutions like Pic2Plate.

In recipe recommendation systems, these traditional methods have evolved to support more dynamic interactions. Initially, recommendation systems relied on static datasets, where recipe recommendations were generated from pre-existing, manually curated databases of ingredients and instructions. However, as food-related content has grown online, some researchers have expanded these systems by connecting them to continuously updated online sources, enabling the recommendation systems to pull from a live, ever-growing collection of recipes [2,3,7]. This connection to online data sources ensures that recommendations are timely and relevant, as new recipes become accessible for recommendation as soon as they are published.

With advancements in computer vision, especially deep learning, recommendation systems for recipes have made significant strides by integrating image recognition capabilities. Modern recipe recommendation frameworks can now use deep learning models to analyze food images, identifying ingredients within the image and generating corresponding recipe recommendations. This approach allows users to find recipes directly by uploading an image of their available ingredients. By transforming visual data into meaningful recipe recommendations, image-based systems have broadened accessibility, enabling users to explore recipes without needing extensive textual input [4,26,27,37].

These advancements in recipe recommendation systems reflect an ongoing shift towards more interactive, adaptable, and user-friendly technologies [11]. With deep learning models capable of both ingredient recognition and recipe retrieval, these systems are closing the gap between ingredient availability and recipe discovery, offering a seamless experience that bridges user needs with relevant culinary suggestions.

## 3. Methodology

**Figure 1:**
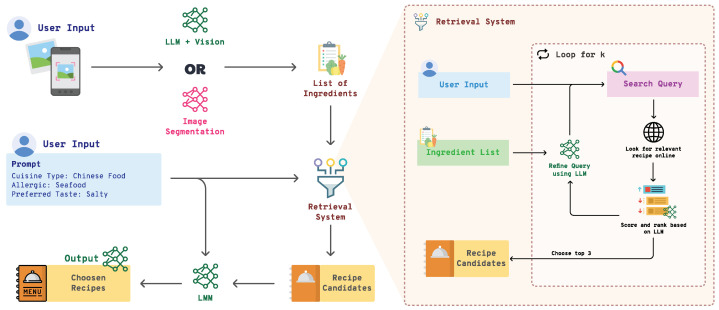
Flowchart of the Pic2Plate System: The user provides an image and preferences (e.g., cuisine type, allergies), which are processed through ingredient recognition. The detected ingredients and user preferences are fed into the RAG system to retrieve and recommend personalized recipes.

The Pic2Plate algorithm is designed to generate personalized recipe recommendations by combining advanced image recognition with natural language processing to interpret user preferences and available ingredients. This methodology is divided into two main parts (Figure 1: Image-to-Text Conversion and Ingredient-Based Recipe Retrieval and Personalization). In the first part, Pic2Plate captures user-provided images, using either direct image recognition or a Vision-Language Model (VLM) to identify ingredients, even in complex scenes. The detected ingredients are then structured as a text-based list, preparing them for the next phase. The second part of the methodology focuses on transforming these ingredients, along with user preferences, into personalized recipe recommendations. By leveraging Large Language Model (LLM)-driven query generation, targeted recipe retrieval, and adaptive recipe adjustments, Pic2Plate ensures that the recommended recipes closely match the user’s dietary needs, available ingredients, and flavor preferences. This approach allows Pic2Plate to deliver highly relevant and customizable recipes, making it a robust, user-centric recommendation system.

### 3.1. Image to Text Conversion

The Pic2Plate framework begins with input from a smartphone camera, a widely accessible and reliable sensor for capturing ingredient images. This choice ensures that the system is compatible with devices that users already possess, enhancing its usability and reducing the need for specialized hardware. By leveraging the smartphone’s camera as the primary input, Pic2Plate simplifies the ingredient detection process, making advanced recipe recommendations available to anyone with a modern mobile device.

The Image-to-Text Conversion phase in the Pic2Plate system is critical for transforming user-provided images of ingredients into a structured list suitable for the recipe retrieval process. In this phase, we explore two approaches for ingredient detection from user images: Image Recognition (via FoodSAM) [25] and Vision-Language Model (VLM)-Assisted Ingredient Detection [14,15]. Together, these dual strategies accurately capture and interpret ingredients, accommodating both simple and complex visual inputs.

In the Pic2Plate framework, visual inputs are categorized as either simple or complex based on the difficulty of ingredient recognition. Simple visual inputs typically feature clear, single, or easily identifiable ingredients presented with good lighting, plain backgrounds, and minimal visual clutter. For example, a photo of a single apple on a white surface or a bunch of carrots on a countertop would be considered simple inputs. In contrast, complex visual inputs contain multiple, overlapping, or partially obscured ingredients, often captured in poor lighting conditions or with busy backgrounds, such as the inside of a cluttered refrigerator or a mixed salad with various components. To handle this variability, Pic2Plate employs FoodSAM for simpler cases where precise segmentation is straightforward. For more complex scenarios, a Vision-Language Model (VLM) is used to interpret the context and relationships between ingredients, even when they are overlapping or partially hidden. This combined approach ensures that Pic2Plate can accurately detect and identify ingredients across a wide range of visual complexities, making the system robust and adaptable to real-world conditions.

The first approach utilizes FoodSAM, a specialized segmentation model developed for precise ingredient recognition in food images. Built upon the Segment Anything Model (SAM), a foundational model in computer vision for image segmentation, FoodSAM is specifically tailored to recognize and segment food items within images. Unlike general-purpose segmentation models, FoodSAM has been trained or fine-tuned on large, food-specific datasets, allowing it to accurately identify individual ingredients, even when they appear in various forms or arrangements (e.g., whole, sliced, or mixed). By leveraging FoodSAM’s segmentation capabilities, Pic2Plate can detect multiple ingredients within a single image, visually isolating and labeling each component. This targeted segmentation approach provides a detailed and structured ingredient list, enabling the system to capture complex ingredient configurations that traditional image recognition models might struggle with.

FoodSAM excels at pixel-level segmentation, isolating each ingredient within a complex food image, defining boundaries, and providing precise ingredient masks. This capability is especially useful in scenarios where ingredients are visually similar or overlapping, such as in salads, stir-fries, or mixed dishes. By leveraging FoodSAM, Pic2Plate achieves detailed ingredient detection, breaking down visually complex dishes into a structured list of recognizable ingredients, which is essential for generating accurate and relevant recipe recommendations.

The Vision-Language Model (VLM) offers an alternative approach to ingredient detection in the Pic2Plate system by providing a holistic, context-based method for interpreting food images. Unlike FoodSAM, which performs precise, pixel-level segmentation, the VLM generates descriptive captions based on its understanding of the entire scene. Models like CLIP or GPT-4o combine visual and linguistic information to interpret images in a way that resembles human perception, going beyond traditional image recognition, which typically focuses on classifying individual objects. This multimodal approach allows VLMs to leverage large datasets of paired images and text, training them not only to recognize objects but also to understand how each object is contextually related to others within the scene. This capability enables the VLM to produce accurate, context-aware descriptions of complex food images, enhancing Pic2Plate’s ingredient detection process.

The core functionality of a VLM lies in its ability to process visual input and output descriptive language that accurately reflects the contents and context of an image. For example, when presented with an image of a complex dish where ingredients may overlap or be partially obscured, a VLM can recognize each ingredient based on its visual features and spatial relationship with surrounding items. By interpreting the scene as a whole, the VLM generates a natural language description that lists the ingredients in a way that considers their arrangement and interaction within the dish.

Using a VLM in the Image-to-Text Conversion phase of Pic2Plate has distinct advantages. Firstly, it enhances the system’s ability to interpret visually complex or cluttered scenes, where precise ingredient boundaries are difficult to define. For instance, in a salad image where ingredients are mixed, the VLM can recognize elements like “lettuce, tomatoes, and cucumbers” without needing to explicitly segment each item. This makes the VLM effective in scenarios where traditional segmentation might misclassify or miss ingredients due to overlaps or occlusions. Another key reason for using a VLM is its flexibility in recognizing ingredients presented in non-standard or mixed states. Since the VLM is trained to interpret relationships and context, it can infer what certain food items are even when they appear alongside other ingredients or in altered forms (such as cooked versus raw). This contextual understanding, learned through large-scale image–text datasets, gives the VLM an edge in capturing the essence of complex dishes and identifying ingredients in ways that pure image recognition or segmentation models might struggle with.

To further enhance the accuracy and adaptability of ingredient detection, Pic2Plate will explore a combined approach that integrates outputs from both FoodSAM and the Vision-Language Model (VLM). In this hybrid method, the VLM receives the segmented ingredient data from FoodSAM as an additional input, allowing it to incorporate FoodSAM’s precise segmentation information into its contextual understanding of the scene.

In practice, this means that FoodSAM first performs segmentation on the image, isolating and labeling distinct ingredients at the pixel level. These segmented ingredient masks are then fed to the VLM, which uses them to refine its contextual captioning. By anchoring its descriptive language generation on the segmented output from FoodSAM, the VLM can produce more accurate and coherent ingredient lists, even in complex images. This combined approach leverages the strengths of both models—the FoodSAM exact boundary recognition and the VLM holistic scene interpretation—enabling Pic2Plate to handle intricate food layouts and diverse ingredient presentations more effectively.

With this integration, Pic2Plate gains a layered analysis of the image, where segmented data reinforce and refine the language model’s output. This synergy between FoodSAM and the VLM ensures that Pic2Plate can robustly identify and interpret ingredients across a wide range of image complexities, providing a highly accurate and structured ingredient list as input for the next stage of recipe retrieval and personalization.

**Figure 2:**
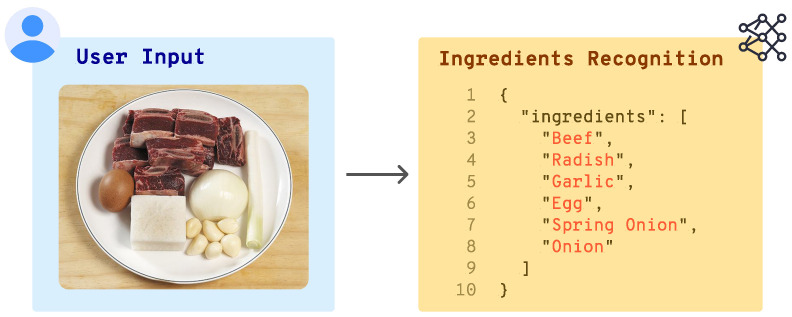
Example of a JSON output from the Pic2Plate system, showing a structured list of detected ingredients based on the user’s image input.

After processing the image with either FoodSAM or the VLM, the identified ingredients are compiled into a structured text file in JSON format (Figure 2). This standardized output format serves as the input for the next phase, Ingredient-Based Recipe Retrieval and Personalization, ensuring that the ingredient list is ready for subsequent retrieval steps. By combining the FoodSAM segmentation precision with the VLM contextual captioning capabilities, Pic2Plate can accurately detect and interpret ingredients from a wide range of visual scenarios, making it adaptable to diverse user inputs.

### 3.2. Retrieval-Augmented Generation System

The Pic2Plate system translates user inputs, consisting of both ingredient images and specific preferences, into personalized recipe recommendations through a structured, multi-step process.

The methodology is divided into four key stages: Query Generation, Recipe Retrieval, Recipe Refinement Using LLM, and Output of Final Recommendations(Algorithm 1). The system integrates the user’s textual preferences (such as dietary restrictions, cuisine types, and flavor preferences) with the list of ingredients detected from the input image. This combined information is used in conjunction with a Large Language Model (LLM) to generate multiple query variations, each designed to capture different combinations of ingredients and preferences. By broadening the search scope through this multi-query approach, the system increases the likelihood of retrieving a diverse and relevant set of recipes from external sources.
**Algorithm 1** Pic2Plate recipe recommendation process.1:**Input:** User query *U*, List of ingredients2:**Output:** Personalized recipe recommendations3:▷ **Query Generation**4:      Feed *U* and ingredients into the LLM5:      Generate multiple queries $Q=\{q_{1},q_{2},\ldots ,q_{n}\}$ based on *U* and ingredients6:▷ **Recipe Retrieval**7:      Retrieve relevant recipes $R_{i}=\left\{r_{1},r_{2},\ldots ,r_{m}\right\}$ from Algorithm 28:▷ **Recipe Refinement Using LLM**9:      Feed $R_{i}$ to the LLM10:        Select top *n* relevant recipes11:        Use LLM to refine and adjust recipes for personalization12:▷ **Output Final Recommendations**13:      Present refined recipes to user

**Figure 3:**
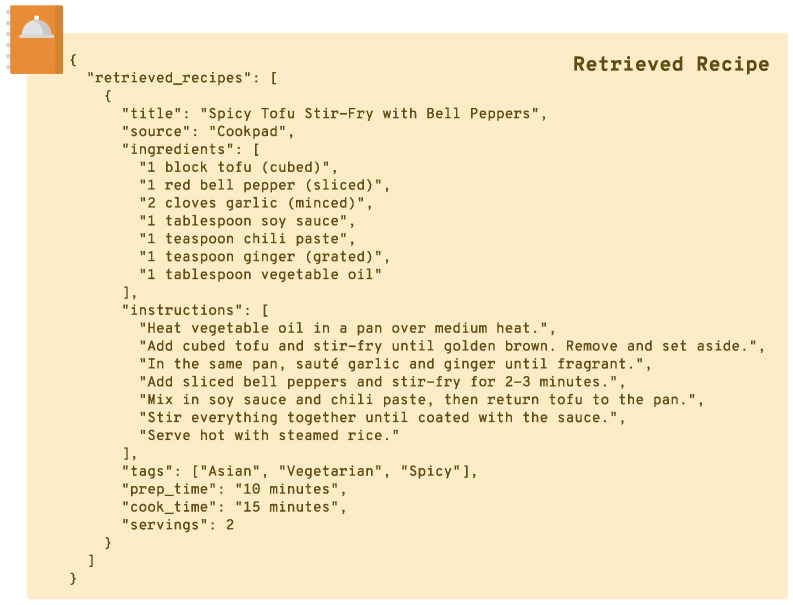
Visualization of a retrieved recipe using the system. The figure shows metadata (e.g., source, tags, preparation, and cooking time), a list of ingredients, and detailed cooking instructions.

In the **Recipe Retrieval** stage, each generated query is sent to external recipe sources online. For each query, the system retrieves a set of recipes that closely align with the input ingredients and user preferences. This retrieval process ensures that Pic2Plate’s recommendations are grounded in real recipes, forming a practical foundation for further personalization. The retrieved recipes include ingredients, preparation steps, and other relevant details, which are collected as an initial pool of options (Figure 3).

Building upon the initial retrieval of recipes, the system proceeds to extract detailed information for each recipe in the pool. This process involves visiting the links associated with each retrieved recipe to gather their ingredients, preparation steps, and other metadata. For example, a retrieved recipe titled “Spicy Garlic Tofu” might yield a list of ingredients such as “tofu, garlic, chili paste, soy sauce” and instructions like “fry tofu until golden” or “mix garlic with chili paste”. These details form the foundation for evaluating the relevance of each recipe to the user’s input.

The **Pic2Plate Retrieval Process** (Algorithm 2) is a systematic approach to fetch, evaluate, and refine recipe recommendations based on user-provided inputs, such as a query and a list of ingredients. This process ensures that the system retrieves and ranks recipes relevant to the user’s preferences, using an iterative mechanism for query refinement with a Large Language Model (LLM), denoted as M.
**Algorithm 2** Pic2Plate retrieval process.1:**Initialize:** Set current query $Q\leftarrow Q_{0}$, iteration counter k←0, LLM model M2:**Set:** Maximum iterations $K_{\max}$, relevance threshold τ3:**while** $k<K_{\max}$ **do**4:    ▷ **Search Recipes:**5:    Use *Q* to query external sources and retrieve recipes $R=\{r_{1},r_{2},\ldots ,r_{n}\}$6:    ▷ **Fetch Recipe Details:**7:    **for** r∈R **do**8:        Extract detailed information: ingredients $I_{r}$, instructions $S_{r}$9:    **end for**10:    ▷ **Evaluate Recipes:**11:    **for** r∈R **do**12:        Generate relevance score using M: score(r)∈[0,10]13:    **end for**14:    Rank recipes by score(r)15:    **if** any recipe *r* satisfies score(r)≥τ **then**16:        **Output:** $R_{\mathrm{final}}\leftarrow$ top *n* recipes from ranked R17:        **Stop**18:    **end if**19:    ▷ **Refine Query:**20:    Use M to refine *Q* based on R, I, and $Q_{0}$21:    Update current query: $Q\leftarrow Q_{\mathrm{refined}}$22:    Increment iteration counter: k←k+123:**end while**24:**Output:** $R_{\mathrm{final}}\leftarrow$ top *n* recipes from the last ranked R

The process begins with the **initialization step**, where the system sets the current query *Q* to the user-provided query $Q_{0}$, initializes an iteration counter k=0, and specifies key parameters, including the maximum number of iterations $K_{\max}$ and a relevance threshold τ. The LLM model M is also loaded during this step, which will later be used for scoring and refining recipes.

In the **Search Recipes** phase, the system uses the query *Q* to query external recipe sources (e.g., Cookpad or Food.com). The result of this query is a collection of recipes $R=\{r_{1},r_{2},\ldots ,r_{n}\}$, each containing basic information such as the recipe title and link. For example, if Q= “spicy tofu stir-fry”, the system might retrieve recipes such as “Spicy Garlic Tofu” and “Ginger Soy Tofu Stir-Fry”.

Next, in the **Fetch Recipe Details** phase, the system iterates over each retrieved recipe r∈R to extract detailed information. This includes the recipe’s ingredients $I_{r}$ and cooking instructions $S_{r}$. For example, for the recipe “Spicy Garlic Tofu”, the extracted ingredients might include “tofu, garlic, soy sauce, chili paste”, while the instructions may describe steps like “fry tofu” or “mix garlic and chili paste”.

The **Evaluate Recipes** phase then uses the LLM M to score the relevance of each recipe *r*. The LLM generates a relevance score score(r) ranging from 0 to 10, based on the overlap between the recipe’s ingredients and the user-provided ingredients, as well as the semantic alignment of the instructions with the query. Recipes are then ranked by their scores, ensuring the most relevant ones are prioritized. For instance, a recipe with “tofu, garlic, chili paste” might score higher than one lacking chili paste if “spicy” was emphasized in the query.

If any recipe in R meets or exceeds the relevance threshold τ, the process outputs the top *n* ranked recipes as $R_{\mathrm{final}}$ and terminates. However, if no recipe satisfies this condition, the **Refine Query** phase is triggered. The LLM generates a refined query $Q_{\mathrm{refined}}$ based on the initial query $Q_{0}$, the retrieved recipes R, and the user-provided ingredients I. For example, if the original query “spicy tofu stir-fry” yields irrelevant results, the refined query might simplify to “tofu garlic chili”.

The system then updates *Q* with $Q_{\mathrm{refined}}$, increments the iteration counter *k*, and repeats the process, beginning with a new search using the refined query. This iterative mechanism continues until a relevant recipe is found or the maximum number of iterations $K_{\max}$ is reached.

Finally, if no recipe meets the relevance threshold after all iterations, the system outputs the best available recipes from the last ranked R. This retrieval process ensures that Pic2Plate provides the most relevant and personalized recipe recommendations possible, adapting dynamically to the user’s inputs and the results of each iteration. For example, starting with a complex query and iteratively refining it ensures that even ambiguous or incomplete inputs yield meaningful results.

Once the recipes are retrieved, the system proceeds to the Recipe Refinement stage, where the LLM further customizes the results to enhance personalization. The LLM filters and adjusts the retrieved recipes based on the user’s preferences and the specific relevance of each recipe. During this refinement process, the LLM ranks the recipes and selects the top *n* most relevant ones, making adjustments to better align with the user’s dietary needs, ingredient availability, and taste preferences. This may involve suggesting ingredient substitutions, modifying quantities, or tailoring preparation instructions. This step ensures that the final recommendations are not only relevant but also uniquely suited to the user’s specific requirements.

In the final stage, the refined recipes are presented to the user as personalized recommendations. These recipes, having undergone both retrieval and LLM-based adjustments, are now highly relevant and tailored to the user’s input. Each recipe is displayed with a comprehensive list of ingredients and detailed instructions that reflect any modifications made during the refinement process. This structured methodology allows Pic2Plate to provide adaptable, user-centric recipe recommendations, making it a versatile solution for personalized culinary guidance based on available ingredients and individual preferences.

## 4. Evaluation

We evaluate the performance of a recommendation system designed to streamline meal planning and enhance the user experience. By leveraging an extensive online recipe dataset, we customize the system’s recommendations based on user preferences, available ingredients, and dietary requirements. Each recipe in the dataset is structured with key elements, including a title, introduction, ingredient list, and detailed preparation steps. By integrating this information with a Large Language Model, we generate tailored outputs. Our evaluation focuses on two critical aspects: the accuracy and reliability of ingredient detection, and the relevance of the recommended recipes. Furthermore, we conduct a survey to measure the effectiveness of this application based on user feedback regarding recipe recommendations. Through these analyses, we aim to demonstrate the system’s effectiveness in delivering precise and user-aligned meal suggestions.

### 4.1. Ingredient Detection Performance

We evaluate the Pic2Plate framework with mixed images from widely distributed smartphones and tablets camera and images from online sources. For the purpose of research, we conceal the model name (Table 1). These devices are selected to represent a diverse range of camera capabilities and configurations, ensuring the framework’s adaptability across various hardware conditions.

The varied focal lengths, apertures, and pixel sizes ensure the framework’s robustness in handling diverse imaging conditions, from well-lit environments to challenging scenarios with mixed lighting. By leveraging the imaging power of modern smartphones, Pic2Plate demonstrates its practicality and accessibility, making personalized recipe recommendations more attainable for a broad audience.

We evaluate the results and analyze the performance of four models used for food-related object recognition: FoodSAM [25], Swin-T [38], LLaMA 3.2 Vision 90B, and GPT-4o. FoodSAM integrates the Segment Anything Model, a semantic segmentation module, and an object detector, enabling it to not only segment objects in images but also categorize them by matching segmented outputs with results from the Segment Anything Model, which are further refined by the object detector. Trained on benchmarks such as UECFoodPix Complete [39] for segmentation masks and FoodSeg103 [6] for food image segmentation, FoodSAM demonstrates a comprehensive approach to food-related object recognition. Similarly, Swin-T employs self-attention-based Swin Transformer blocks with hierarchical representations to extract features effectively, supporting accurate food segmentation tasks. Both models were trained using the FoodSeg103 dataset.

**Figure 4:**
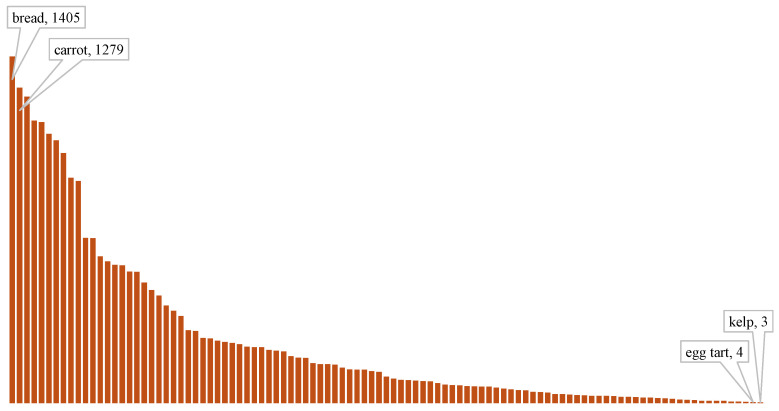
FoodSeg103 dataset class spread.

The FoodSeg103 dataset consists of 7118 images across 103 classes, ranging from vegetables to types of fungi. While it provides detailed food ingredient classes, the dataset suffers from an imbalanced distribution of images per class, which poses challenges in accurately detecting less-represented food categories. This imbalance particularly impacts model performance when identifying rare food types, underscoring the importance of architectural choices and training data diversity in overcoming these challenges (Figure 4).

**Figure 5:**
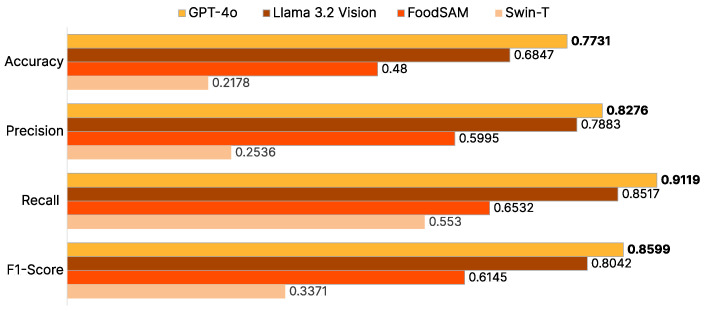
Ingredient detection evaluation chart (best performance is highlighted in **bold**).

We use classification evaluation metrics, including **precision**, **recall**, **F1-score**, and **accuracy** (Figure 5). These metrics provide valuable insights into the strengths and weaknesses of each model.

The discussion highlights the comparative performance of these models, focusing on their precision in identifying food items, recall of the human-labeled data, and overall alignment with **human evaluations**.

These metrics, commonly used in classification and object recognition tasks, offer a robust measure of the model’s accuracy and relevance in detecting specific ingredients within an image. However, it is important to note that True Negatives (TN) are not applicable in this project. Since the system is designed to detect only relevant ingredients in an image, the concept of correctly identifying “absent” ingredients does not apply.

Thus, the evaluation focuses solely on True Positives (TP), representing items correctly identified by the model; False Positives (FP), indicating items incorrectly predicted by the model that were not present in the human-labeled data; and False Negatives (FN), denoting items present in the ground truth but missed by the model.

Precision quantifies the proportion of correctly identified ingredients among all predictions made by the model. It evaluates the accuracy of positive predictions, defined as

(1) $$\mathrm{Precision}=\frac{TP}{TP+FP}$$

where TP (True Positives) represents correctly identified ingredients, and FP (False Positives) refers to incorrectly predicted ingredients that are absent in the image. A high precision score indicates the model’s ability to minimize false positives, ensuring reliable predictions.

Recall assesses the model’s capability to detect all relevant ingredients present in the image. It is expressed as

(2) $$\mathrm{Recall}=\frac{TP}{TP+FN}$$

where FN (False Negatives) denotes ingredients that are present in the image but missed by the model. High recall reflects the model’s effectiveness in reducing undetected ingredients and comprehensively identifying relevant instances.

Accuracy evaluates the proportion of correctly classified instances across all predictions. While accuracy is a straightforward measure of overall performance, it can be less informative for imbalanced datasets, where certain classes are underrepresented. It is calculated as

(3) $$\mathrm{Accuracy}=\frac{TP+TN}{TP+TN+FP+FN}$$

Here, TN (True Negatives) represents instances that are correctly identified as irrelevant. Accuracy is most effective when the costs of false positives and false negatives are similar.

The F1-score, defined as the harmonic mean of precision and recall, offers a balanced evaluation of the model’s performance, particularly in scenarios with uneven class distributions:

(4) $$F1\text{-}\mathrm{score}=2\times \frac{\mathrm{Precision}\times \mathrm{Recall}}{\mathrm{Precision}+\mathrm{Recall}}$$

This metric ensures a comprehensive understanding of the trade-off between precision and recall, making it invaluable for evaluating object recognition models.

In the evaluation of model performance, GPT-4o achieved the best results across all metrics, outperforming the other models tested. It achieved a precision of 0.83, recall of 0.91, F1-score of 0.86, and accuracy of 0.77. Following closely, LLaMA 3.2 Vision recorded a precision of 0.79, recall of 0.85, F1-score of 0.80, and accuracy of 0.68. FoodSAM, with its integration of detection and segmentation modules and additional datasets, showed moderate performance with a precision of 0.60, recall of 0.65, F1-score of 0.61, and accuracy of 0.48. In contrast, Swin-T, trained solely on the imbalanced FoodSeg103 dataset, faced challenges and recorded a precision of 0.25, recall of 0.55, F1-score of 0.34, and accuracy of 0.22.

The imbalanced nature of FoodSeg103 posed significant challenges for Swin-T, which relies solely on this dataset, whereas FoodSAM mitigated some of these issues by leveraging additional datasets and integrating detection and segmentation modules. These enhancements reduced false detections, allowing FoodSAM to outperform Swin-T. However, when compared with vision LLMs like GPT-4o and LLaMA 3.2 Vision, FoodSAM fell short. Vision LLMs, trained on diverse and generalized datasets, benefit from advanced reasoning capabilities, enabling them to better capture the context of images and the objects within them. This diverse training and contextual understanding make vision LLMs more effective in accurately identifying food-related objects and ingredients across a broader range of scenarios.

While ChatGPT appeared as the top-performing model across all metrics, LLaMA 3.2 Vision, with its 11 billion parameters, showcased impressive competitiveness against OpenAI’s state-of-the-art model, which in the best of our knowledge, operates with a significantly larger parameter count. In contrast, FoodSAM struggled to generalize when presented with previously unseen data, highlighting its reliance on training-set familiarity and underscoring the challenges of accurately detecting specific ingredients in more diverse scenarios. This highlights the importance of model scalability and robustness, as larger models like ChatGPT demonstrate a clear advantage in handling diverse and previously unseen inputs. However, the competitiveness of LLaMA 3.2 Vision suggests that well-optimized architectures can bridge the performance gap even with comparatively fewer parameters.

### 4.2. Recipe Relevance Measurement

**Figure 6:**
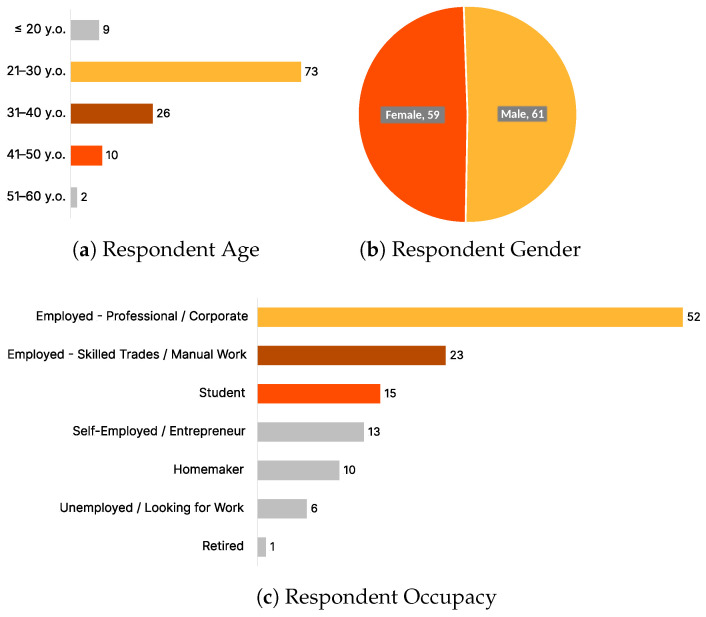
The Respondent Demography.

To evaluate the recipe relevance of Pic2Plate, a survey was conducted with 120 participants who provided feedback on the model’s performance. The survey covered the second components of the Pic2Plate methodology: personalized recipe recommendation using large language models (LLMs) and Retrieval-Augmented Generation (RAG). The respondents represented a diverse demographic background in terms of gender, age, and occupation (Figure 6).

The gender distribution of the respondents was nearly balanced, with 59 females and 61 males participating in the study. Regarding age, the majority of respondents were between 21 and 30 years old (73 participants), followed by 31–40 years old (26 participants). Smaller groups included those aged ≤20 (9 participants), 41–50 (10 participants), and 51–60 (2 participants). The occupational distribution was equally diverse: 52 professionals in corporate settings made up the largest group, followed by students (23), and smaller groups such as self-employed entrepreneurs (15), homemakers (10), and retired individuals (1). This demographic spread ensures comprehensive feedback across varied user needs and experiences.

The survey involved five distinct example sets (Appendix B), each requiring participants to assess models identified only by aliases—Model A (LLaMA 3.1 70B + RAG), Model B (LLaMA 3.1 70B only), and Model C (GPT-4o + RAG)—to maintain objectivity. For each set, participants were asked to rank the models based on two key questions: Which model performed the best? And Which model performed the worst? (Appendix A)

The rankings were analyzed using the Bradley–Terry method, which provides a statistically robust mechanism for comparing paired preferences and computing model rankings [40,41,42,43]. This method assumes that the probability of one model *i* being preferred over another model *j* is proportional to their respective strengths $s_{i}$ and $s_{j}$. Specifically, the probability is expressed as

(5) $$P\left(i\;\mathrm{beats}\;j\right)=\frac{s_{i}}{s_{i}+s_{j}}$$

where $s_{i}>0$ and $s_{j}>0$ represent the strengths of models *i* and *j*, respectively.

Given a set of pairwise wins $w_{ij}$, which denotes the number of times model *i* is preferred over *j*, the likelihood of observing the data is modeled as

(6) $$L=\prod_{i,j\in \mathrm{models}}{\left(\frac{s_{i}}{s_{i}+s_{j}}\right)}^{w_{ij}}$$

Taking the logarithm of the likelihood to simplify computation, we obtain the log-likelihood function:

(7) $$\log L=\sum_{i,j\in \mathrm{models}}w_{ij}\cdot \log\left(\frac{s_{i}}{s_{i}+s_{j}}\right)$$

The Bradley–Terry model estimates the strengths $s_{i}$ for all models by minimizing the negative of the log-likelihood logL. This is achieved using numerical optimization techniques, such as the L-BFGS-B algorithm [43]. Once the strengths $s_{i}$ are estimated, they are normalized to ensure that the sum of all strengths equals 1:

(8) $$\hat{s_{i}}=\frac{s_{i}}{\sum_{j}s_{j}}$$

**Figure 7:**
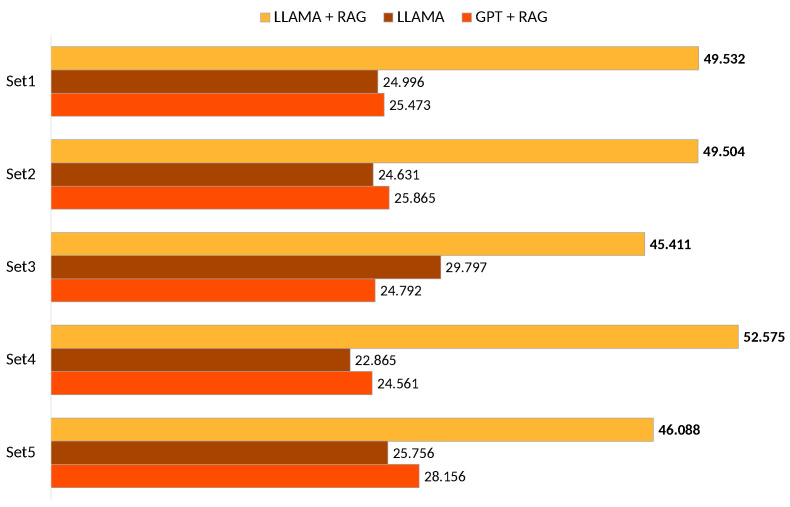
Comparison of model rankings across five sets using Bradley–Terry analysis (best model is highlighted in **bold**).

The models are then ranked based on their normalized strengths $\hat{s_{i}}$ (Figure 7), with higher strengths indicating better performance. We calculate the pairwise wins $w_{ij}$ from survey data as follows:

- The number of “Best Votes” contributes to wins for the selected model.
- The number of “Worst Votes” contributes to losses for the selected model.

The results of the Bradley–Terry analysis reveal notable differences in the perceived performance of the models. Across all five sets (Figure 6), Model A (LLaMA + RAG) consistently achieved higher ranks compared with the alternatives, Model B (LLaMA only) and Model C (GPT-4o + RAG). The aggregate rankings demonstrate a clear preference for the integration of Retrieval-Augmented Generation (RAG) with LLaMA in delivering personalized recipe recommendations. These findings highlight the benefits of combining RAG frameworks with advanced language models to enhance user satisfaction and relevance in recipe suggestions. LLaMA only shows competitive performance, often nearing GPT-4o + RAG but falls short of LLaMA + RAG. These results highlight that combining RAG with a well-optimized model like LLaMA significantly enhances recipe recommendations. The gap between LLaMA + RAG and GPT-4o + RAG suggests GPT-4o struggles to effectively leverage RAG, emphasizing the importance of choosing the right base model.

**Figure 8:**
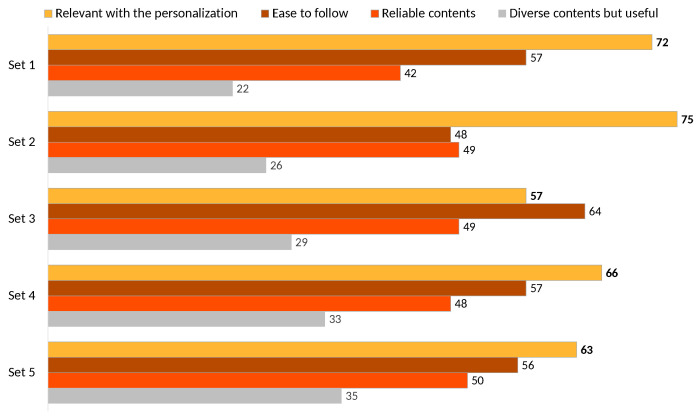
User preferences across evaluation dimensions for Pic2Plate models.

Participants also evaluated the models across several dimensions, including relevance with personalization, ease of following recommendations, content reliability, and diversity of suggested recipes (Figure 8). Pic2Plate’s framework demonstrates consistently positive evaluations across multiple dimensions. The highest scores were observed in the ‘Relevant with personalization’ category, particularly in Set 2 (75) and Set 1 (72), indicating strong alignment with user preferences. The ‘Ease to follow’ category also received favorable ratings, notably in Sets 1, 3, and 4 (scores of 57, 64, and 57, respectively), showcasing the perceived dependability of the recommendations. While ‘Reliable contents’ scored moderately, with its highest rating in Set 5 (50), it highlights an area for potential improvement. ‘Diverse contents but useful’ exhibited comparatively lower ratings across sets, peaking at 35 in Set 5, suggesting that while users value diversity, it might require further refinement to enhance perceived utility. Overall, the framework excels in relevance and reliability, with some room to optimize reliability and diversity dimensions.

## 5. Conclusions

This paper introduces Pic2Plate, a novel recipe recommendation system that combines Vision-Language Models (VLMs) and Retrieval-Augmented Generation (RAG) to address challenges in ingredient detection and deliver highly personalized recipe recommendations. By leveraging widely available smartphone cameras as sensors and embracing the shift toward digital recipe guidance, Pic2Plate offers a practical and inclusive solution for modern cooking. This accessibility ensures that personalized recipe recommendations are available to a broad user base, empowering individuals to make informed culinary choices with the tools they already have. By integrating precise image segmentation with context-aware generation and leveraging an online retrieval mechanism, the framework provides up-to-date, tailored recipes. This combination of advanced VLM and RAG technologies, alongside real-time retrieval, offers a more dynamic and context-aware approach compared to traditional systems, marking a meaningful step forward in culinary AI. This approach effectively alleviates the burden of cooking preparation, accommodates personalized dietary needs, and mitigates language model hallucination to ensure the best possible recipe suggestions for users.

The evaluation of Pic2Plate demonstrates its effectiveness in both ingredient detection and recipe recommendation. The results highlight the system’s robust performance: GPT-4o achieved the highest precision (0.83), recall (0.91), and F1-score (0.86) in ingredient detection. The human feedback survey confirms that the integration of VLMs and RAG provides significant improvements in relevance, reliability, and ease of use (Figure 8). With the strong results of the ingredient detection module, and supported by the survey findings, these outcomes validate Pic2Plate as a robust and user-centric framework for personalized recipe recommendations. Moreover, this proof highlights the advancement of recipe recommendation frameworks by seamlessly incorporating camera sensors from everyday devices, showcasing the ease of use and accessibility for daily applications. One notable limitation is the reliance on the FoodSeg103 dataset for training, which, while comprehensive with 103 food classes and 7118 images, suffers from imbalanced class distributions. This imbalance may affect the model’s ability to accurately detect less-represented or rare ingredients, potentially limiting its robustness in diverse real-world scenarios. By leveraging Vision-Language Models (VLMs), Pic2Plate enhances its ability to generalize across diverse contexts, as these models integrate visual understanding with contextual reasoning, allowing for more robust performance even with imbalanced training data.

Furthermore, the system’s dependence on internet connectivity restricts usability in offline contexts, and managing rare or region-specific ingredients remains a challenge due to the dataset’s limitations. Looking ahead, there are valuable opportunities for enhancement. Expanding the training datasets to include a broader and more balanced representation of ingredients and cuisines would improve detection accuracy and system versatility. Developing offline functionalities with preloaded recipe databases could address connectivity constraints, while incorporating adaptive learning mechanisms would enable the system to refine its performance over time. Additionally, exploring multi-modal data integration and leveraging user feedback loops would enhance personalization and better cater to the diverse needs of a wider user base, making Pic2Plate a more inclusive and effective tool.

## Figures and Tables

**Table 1:** Specifications of smartphones used for evaluation.

Mobile Device	Camera Sensor	Resolution (MP)	Focal Length (mm)	Aperture (f)
Smartphone 1	Sony, 1.9 µm	12	26	1.5
Smartphone 2	Sony IMX766V, 1.0 µm	50	10	1.8
Smartphone 3	Samsung S5KGN3, 1.0 µm	50	23	1.8
Smartphone 4	Samsung S5K2LD, 1.8 µm	12	24	1.8
Tablet 1	-, 1.8 µm	13	-	1.8
Tablet 2	-, 1.0 µm	13	26	2.0

## Data Availability

The datasets, models, and code used in this research are available upon request from the corresponding author, subject to reasonable conditions. Due to privacy and data sensitivity concerns, these resources are not publicly accessible.
